# Supplementary material for: CXCL12 targets the primary cilium cAMP/cGMP ratio to regulate cell polarity during migration
Source: Nat Commun. 2023 Dec 4;14:8003. doi: 10.1038/s41467-023-43645-w (PMC10695954; doi:10.1038/s41467-023-43645-w)
Supplement: Supplementary file 1 — Supplementary Information [file 41467_2023_43645_MOESM1_ESM.pdf]

Supplementary Information

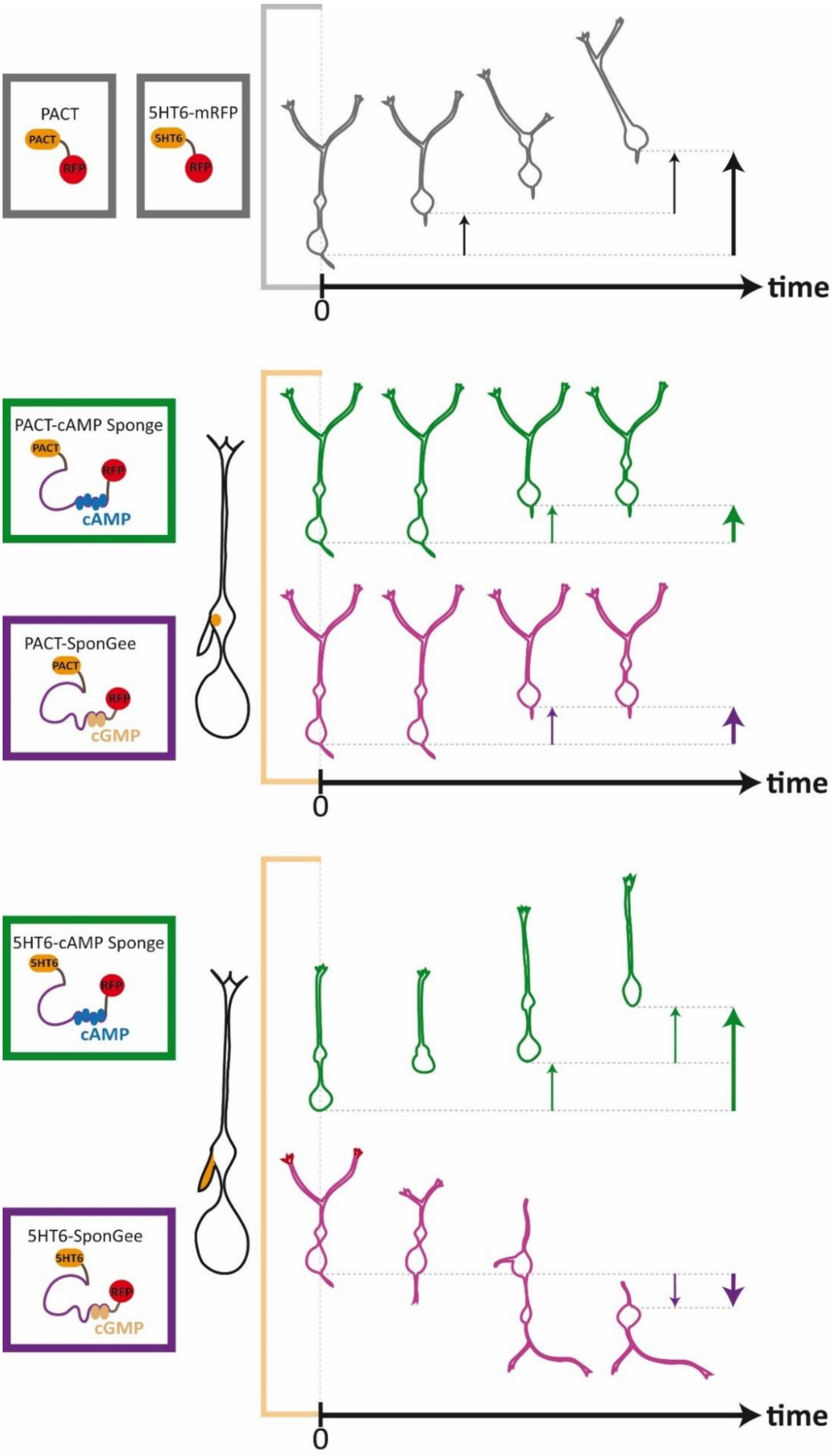

Supplementary Fig. 1: Summary diagram depicting the migratory phenotypes associated with cAMP or cGMP buffering at the centrosome or at the PC compartment. Migrating cortical interneurons electroporated with the control constructs (top panel) rhythmically alternate over time between phases of pause and nucleokinesis in a saltatory mode. Two events of nucleokinesis are depicted on the diagram, each represented by the induced displacement vector (thin arrow). The thick arrow represents the sum of each individual displacement vector. When the cAMP or cGMP scavengers are targeted to the centrosome (middle panel), the polarity of the cell (defined by the nucleus-swelling axis) is unchanged, but the frequency of nucleokinesis is reduced, resulting in increased

pausing times and reduced migration speed. For the same amount of time, only one nucleokinesis event occurs and the final displacement vector is shorter compared to controls, although its directionality is unchanged. Finally, when the scavengers are addressed to the PC (bottom panel), ciliary cAMP or cGMP buffering induces opposite phenotypes on cell polarity and directionality. Ciliary cAMP buffering induces migrating cells to maintain their polarity (i.e., the centrosome moves forward and nucleokinesis occurs within the same leading process) and to reduce branching events at the leading process, resulting in increased directionality. The migration speed is unchanged compared to controls, as highlighted by the same frequency of nucleokinesis events and the final displacement vector, which is of equal length and directionality compared to the control situation. By contrast, cGMP buffering at the PC induces more branching at the soma compartment and frequent changes in cell polarity (i.e., the centrosome moves from one leading process to a newly-formed branch that becomes the new leading process), which results in a decreased directionality and migration speed. As a result, the final displacement vector is not only shorter compared to controls, but it is also inverted.

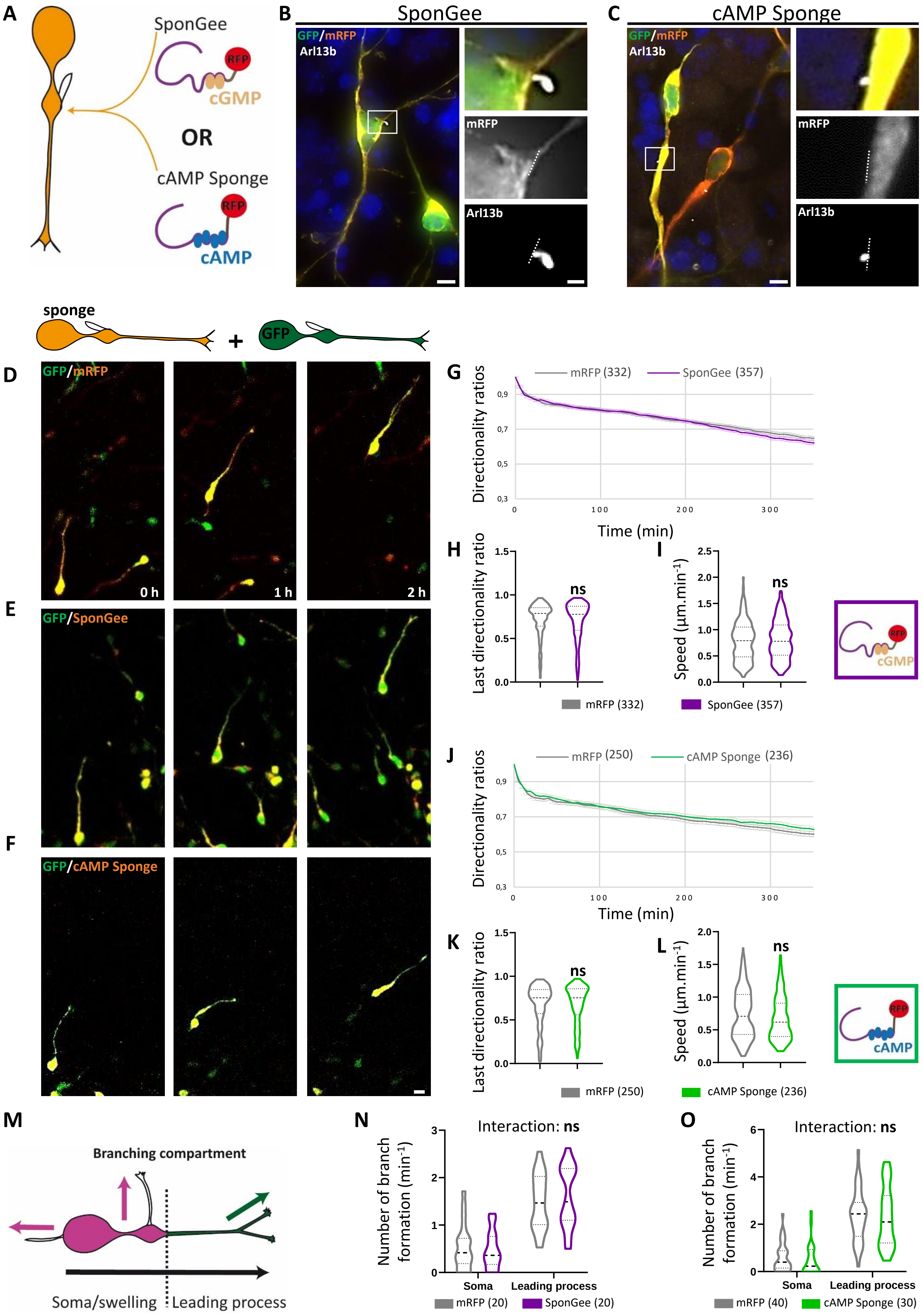

A
SponGee
RFP
cGMP
OR
cAMP Sponge
RFP
cAMP
B
SponGee
GFP/mRFP
Arl13b
mRFP
Arl13b
C
cAMP Sponge
GFP/mRFP
Arl13b
mRFP
Arl13b
sponge
+
GFP
D
GFP/mRFP
0 h
1 h
2 h
E
GFP/SponGee
F
GFP/cAMP Sponge
G
mRFP (332)
SponGee (357)
Directionality ratios
Time (min)
H
Last directionality ratio
ns
I
Speed (µm.min-1)
ns
mRFP (332)
SponGee (357)
RFP
cGMP
J
mRFP (250)
cAMP Sponge (236)
Directionality ratios
Time (min)
K
Last directionality ratio
ns
L
Speed (µm.min-1)
ns
mRFP (250)
cAMP Sponge (236)
RFP
cAMP
M
Branching compartment
Soma/swelling
Leading process
N
Interaction: ns
Number of branch formation (min-1)
Soma
Leading process
mRFP (20)
SponGee (20)
O
Interaction: ns
Number of branch formation (min-1)
Soma
Leading process
mRFP (40)
cAMP Sponge (30)

Supplementary Fig. 2: Expression of the SponGee or cAMP Sponge scavengers in the whole cytoplasm does not affect cortical interneuron directionality or motility. (**A**) Representative scheme of a cortical interneuron and its PC. The mRFP-tagged SponGee or cAMP Sponge scavengers lacking the 5HT6-targeting sequence are addressed to the whole cytoplasm – excluding the cilioplasm. (**B-C**) High magnification of cortical interneurons co-electroporated with a cytoplasmic GFP construct and the SponGee (B) or cAMP Sponge (C) chelators. Cells were immunostained with anti-GFP, anti-RFP and anti-Arl13b antibodies. Notably, when addressed to the whole cytoplasm, the SponGee and cAMP Sponge scavengers fail to enter the Arl13b-positive PC. Insets are higher magnifications of the boxed region on the left. Dotted lines delimitate the border between cytoplasm and cilioplasm. Scale bar, 5 μm; in insets, 1 μm. Cytoplasmic localisation of the constructs was observed in three independent experiments. (**D-F**) Time-lapse recordings of cortical interneurons co-electroporated with the GFP cytoplasmic construct and the RFP control construct (the tag without the sponge; D), SponGee (E) or cAMP Sponge (F). Scale bar, 10 μm. (**G**) Graphical representation of the mean directionality ratios at each time point for the RFP and SponGee conditions. (**H**) Mean directionality ratio after a maximum 350-minute migration period. (**I**) Mean migration speed. (**J**) Graphical representation of the mean directionality ratios at each time point for the RFP and cAMP Sponge conditions. (**K**) Mean directionality ratio after a 350-minute migration period. (**L**) Mean migration speed. (**M**) Schematics of branch quantification in (N,O). Leading process branches (green) initiate milder changes in direction (green arrow) than branches from the soma/swelling compartment (pink; pink arrows). (**N-O**) Mean branching frequency from the soma/swelling and leading process compartments for SponGee- (N) and cAMP Sponge-electroporated cells (O) compared to RFP controls. The number of cells is indicated in the graph legends. P $\leq$ 0.001; ****; ns, non significant. Two-tailed Mann-Whitney test (H,I,K,L). Two-way ANOVA test with Bonferroni's multiple comparison post test (N) Interaction: ns; Genotype effect: ns; Compartment effect: ****. (O) Interaction: ns; Genotype effect: ns; Compartment effect: ****. Error bars are SEM. Source data and p values are provided as a Source data file.

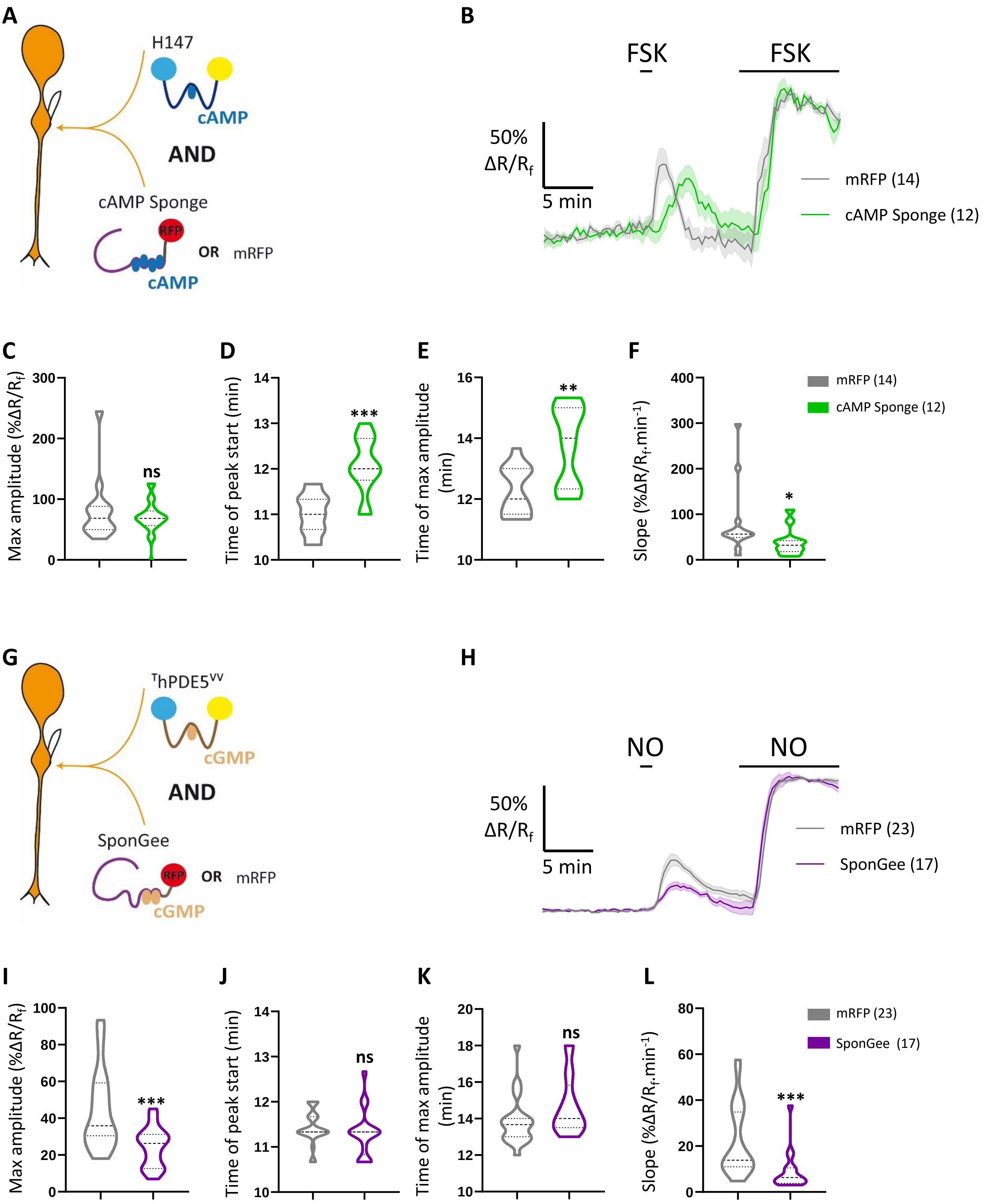

Supplementary Fig. 3: The cAMP Sponge and SponGee scavengers buffer cAMP and cGMP (respectively) in the cytoplasm of migrating cortical interneurons. (**A**) Representative scheme of a cortical interneuron and its PC. The H147 cAMP biosensor and the mRFP-tagged cAMP Sponge scavenger lacking the 5HT6-targeting sequence are co-electroporated and expressed in the whole cytoplasm. (**B**) cAMP Sponge-expressing cells (green trace) exhibit a delayed response to a 20-second forskolin (FSK) pulse as compared to control mRFP-expressing cells (grey trace). A 10-minute forskolin application was applied at the end of the recording to check for cell viability and for biosensor functionality. As expected, the cAMP chelator is saturated by this prolonged exposure. (**C-F**):

Quantification of the maximum amplitude of the CFP:YFP ratio in the eight minutes following the forskolin pulse (C), the time at which the forskolin peak is initiated (D), the time when maximum peak amplitude was measured (E) and of the slope of the forskolin peak (F). (**G**) The ^{T}hPDE5^{VV} cGMP biosensor and the mRFP-tagged SponGee scavenger lacking the 5HT6-targeting sequence are co-electroporated and expressed in the whole cytoplasm. (**H**) SponGee-expressing cells (purple trace) exhibit a reduced response to a 40-second spermine NONOate (NO) pulse as compared to control mRFP-expressing cells (grey trace). A 10-minute NO application was applied at the end of the recording to check for cell viability and for biosensor functionality. As expected, the cGMP chelator is saturated by this prolonged exposure. (**I-L**) Quantification of the maximum amplitude of the YFP:CFP ratio in the ten minutes following the NO pulse (I), the time at which the NO peak is initiated (J), the time when maximum peak amplitude was measured (K) and of the slope of the NO peak (L). The number of analysed cells is indicated in the graph legends. *, $P \leq 0.05$; **, $P \leq 0.01$; ***, $P \leq 0.001$, ns, non significant. Two-tailed Mann-Whitney test (C-F, I-L). Error bars are SEM. Source data and p values are provided as a Source data file.

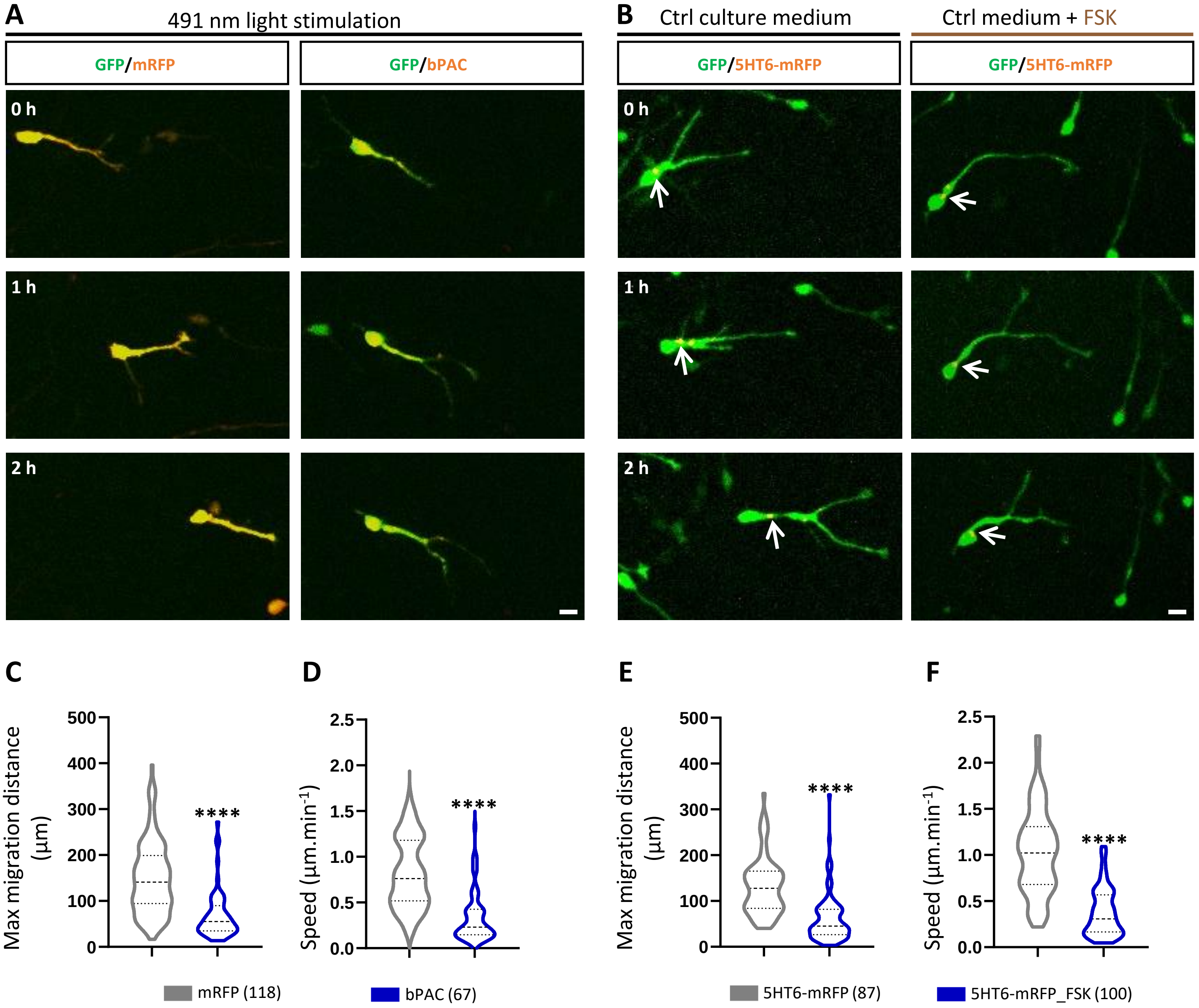

Supplementary Fig. 4: Pharmacologically or optogenetically increased cAMP levels in the whole cytoplasm inhibit the motility of migrating cells. (A) Time-lapse recordings of cortical interneurons co-electroporated with the GFP cytoplasmic construct and the non-addressed RFP (A, left-hand panel) or RFP-tagged light sensitive adenylyl cyclase (bPAC; A, right-hand panel) constructs. bPAC is photo-activated by blue light (491 nm laser) every minute for 2,1 seconds, leading to increased cytoplasmic cAMP production. Scale bar, 10 µm. **(B)** Time-lapse recordings of cortical interneurons electroporated with the 5HT6-mRFP control construct and imaged in the absence (left-hand panel) or presence (right-hand panel) of forskolin (FSK) in the culture medium. Imaging was initiated at the onset of migration. Forskolin was added to the culture medium after five hours, and imaging continued for another ten hours. White arrows point the mRFP-tagged primary cilium. Sacle bar, 10 µm. **(C-F)** Representation of the maximum migration distance achieved over a five-hour period (C,E) and of the mean migration speed (D,F) of the mRFP- and bPAC-electroporated cells (C-D) and of the 5HT6-mRFP electroporated cells prior or after forskolin application (E-F). The number of cells is indicated in the graph legends. $P \leq 0.0001$; ****. Two-tailed Mann-Whitney test (C-F). Source data and p values are provided as a Source data file.

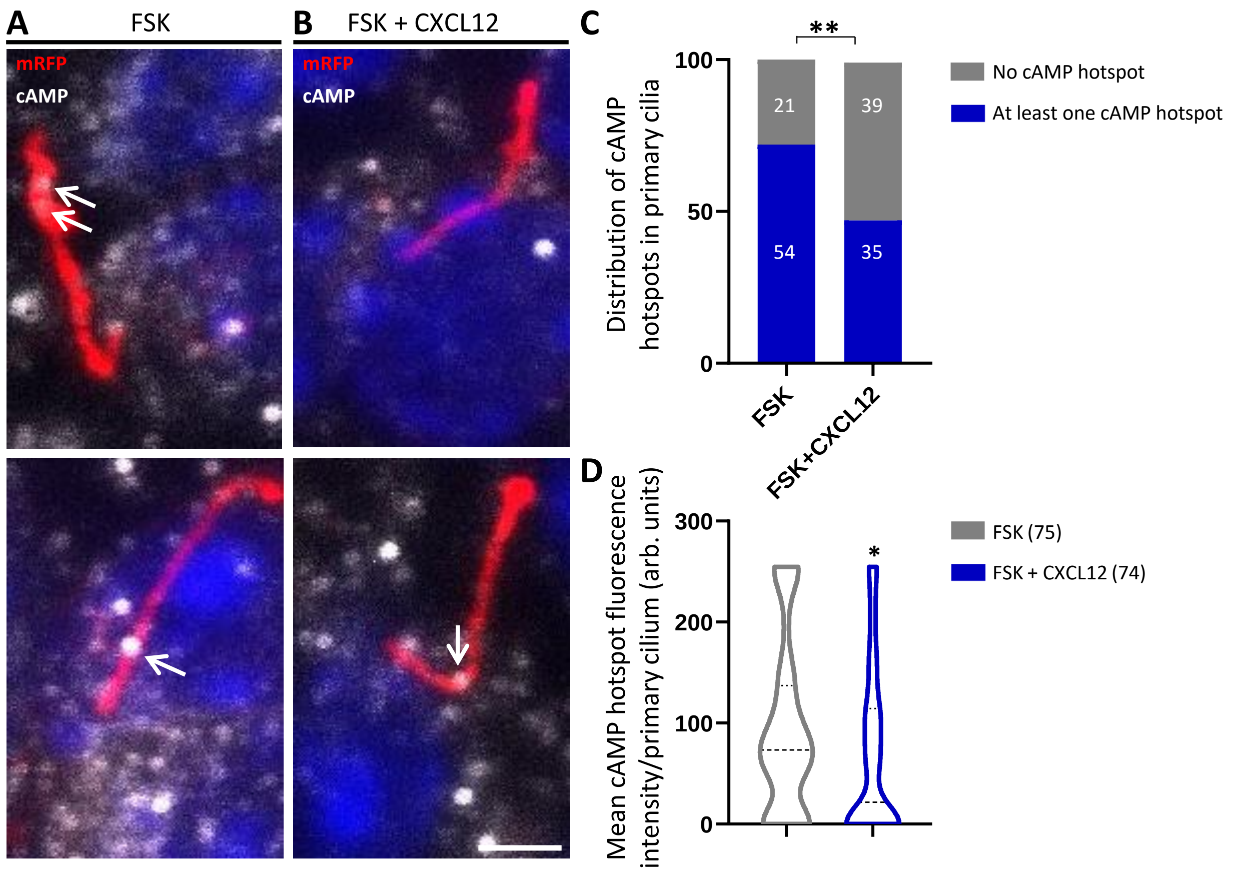

Supplementary Fig. 5: CXCL12 bath application decreases the number and fluorescence intensity of cAMP hotspots detected in cortical interneuron primary cilia. In order to visualise the effect of CXCL12 on cAMP levels within the primary cilium, migrating cortical interneurons were treated for 15 minutes with or without CXCL12 and in the presence of forskolin (FSK), before fixation. **(A-B)** Confocal images of cortical interneuron primary cilia cultured in the absence (left) or presence (right) or CXCL12. Z-stacks were performed so as to image the whole primary cilium, with a step size of 0,5 µm. The punctate cAMP staining observed both in the primary cilia (white arrows) and surrounding cytoplasm is coherent with the cAMP immunostaining pattern observed in prior studies. Only the cAMP hotspots that co-localised with the primary cilium in a same confocal plane were considered as belonging to the primary cilium. Analyses were performed on cells obtained over three independent experiments. Scale bar, 2 µm. **(C)** Distribution of cAMP hotspots in the primary cilia of cultured cortical interneurons. **(D)** Mean fluorescence intensity of the ciliary cAMP hotpots. The number of cells is indicated on the graph (C) or in the graph legends (D). *, $P \leq 0.05$; **, $P \leq 0.01$, ns, non significant. Two-tailed Chi-square test (C) and Two-tailed Mann-Whitney test (D). Source data and p values are provided as a Source data file.

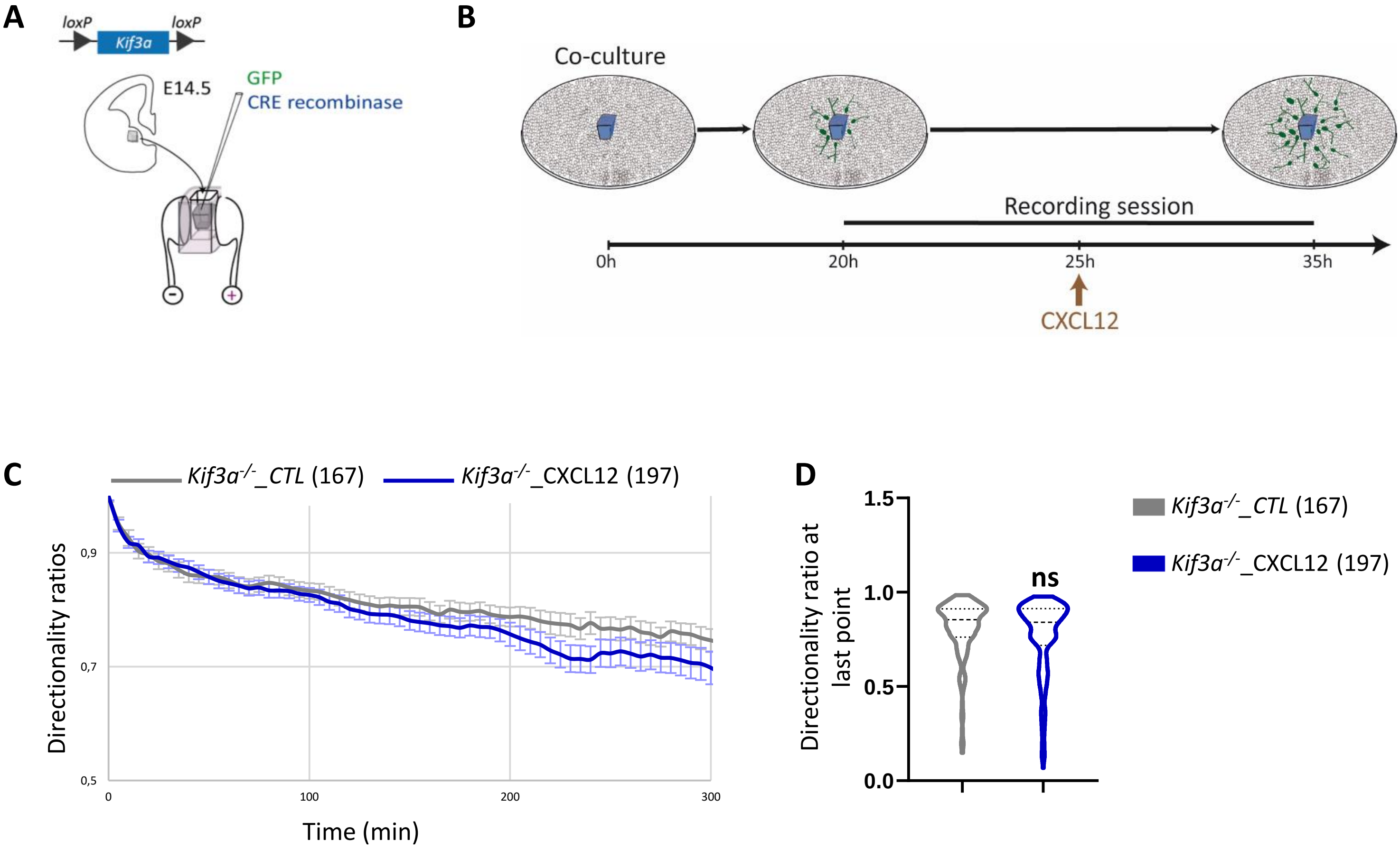

Supplementary Fig. 6: Genetic ablation of the primary cilium compartment abolishes the effect of CXCL12 on the directionality of migrating cortical interneurons. (A) MGEs are dissected from E14.5 *Kif3a*$^{fl/fl}$ mouse embryos and co-electroporated with the cytoplasmic GFP and the pCAGGS-Cre constructs to induce a non-ciliated cell population. **(B)** Representative scheme of the *in vitro* protocol. MGE explants electroporated with GFP and pCAGGS-Cre are co-cultured on dissociated cortical cells. Live imaging starts as cortical interneurons initiate their migration and continues for 10 hours after CXCL12 is added to the culture medium. **(C)** Mean directionality ratios measured at each time point before (grey) or after (blue) CXCL12 application. **(D)** Mean directionality ratio after a maximum 300-minute period. CTL, control; ns, non significant. Two-tailed Mann-Whitney test (D). Error bars are SEM. Source data and p values are provided as a Source data file.

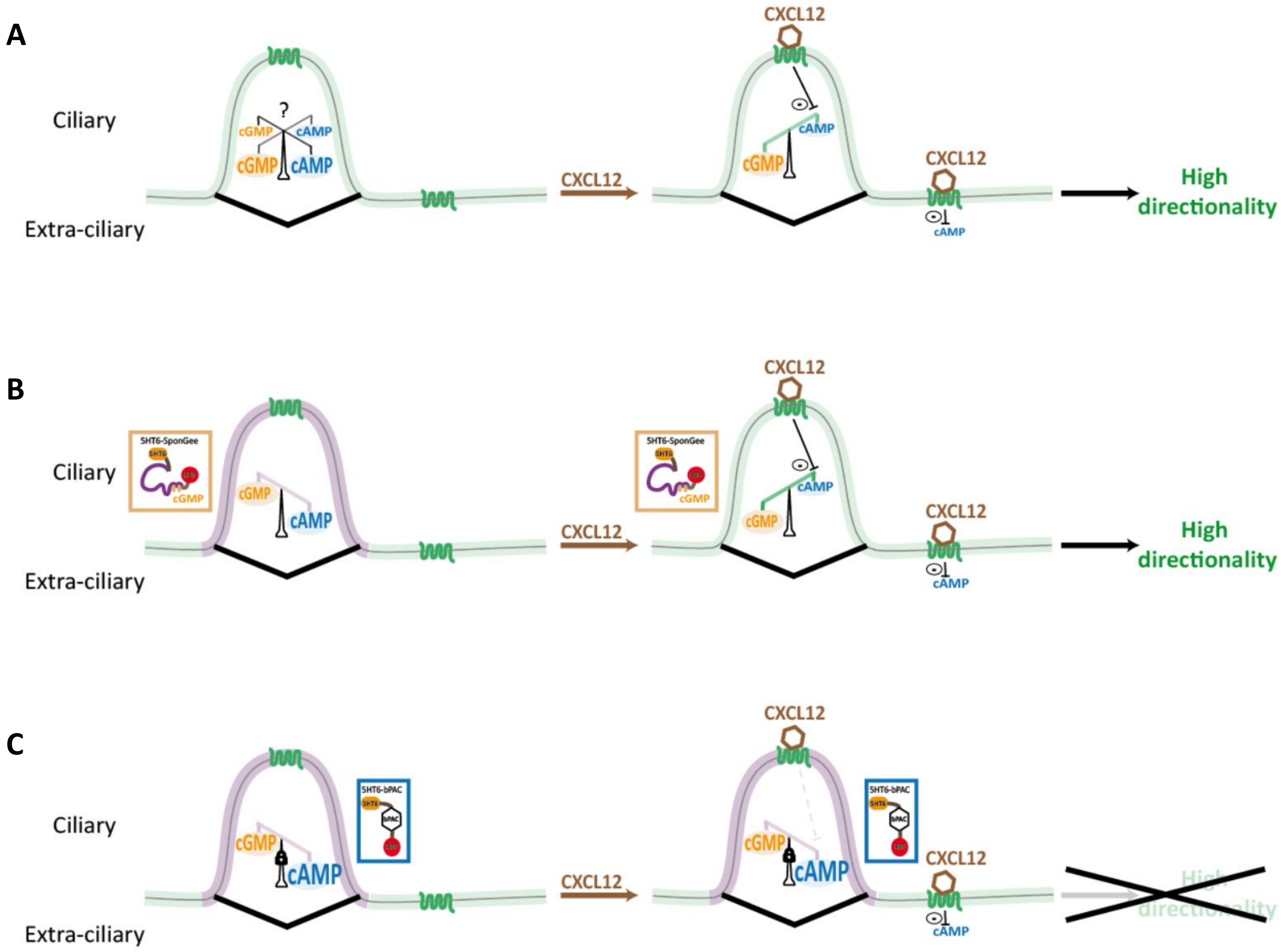

Supplementary Fig. 7: Summary diagram depicting the effect of CXCL12/CXCR4 signalling on the ciliary cGMP/cAMP ratio and on directed cell migration in three experimental conditions analysed in the present study. **(A)** CXCL12 application on migrating cortical interneurons decreases ciliary and extra-ciliary cAMP levels in a CXCR4-dependent manner, resulting in a increased migration directionality. **(B)** In migrating cortical interneurons, ciliary cGMP buffering stabilises the ciliary cGMP/cAMP ratio in a conformation with higher cAMP levels with regards to the cGMP levels. CXCL12 bath application on cortical interneurons electroporated with the 5HT6-SponGee construct induces a reduction in cAMP levels in the cytoplasm of migrating cells but also in their cilioplasm, leading to a reduced ciliary cGMP/cAMP ratio. The cell directionality of migrating cells is increased. **(C)** In migrating cortical interneurons, expression and photo-activation of the primary cilium-targeted bPAC construct increases ciliary cAMP levels. As with the 5HT6-SponGee construct, the ciliary cGMP/cAMP ratio is stabilised in a conformation with higher cAMP levels with regards to the cGMP levels. This low cGMP/cAMP cannot be increased by CXCL12 application through cAMP inhibition: bPAC is a bacterial soluble adenylyl cyclase, which is not affected by mammalian G proteins. Following CXCL12 bath application, cAMP levels are therefore only decreased in the cytoplasm at the exclusion of the ciliosplasm. As a consequence, cell directionality is no longer increased.
