# Supplementary material for: CXCL12 targets the primary cilium cAMP/cGMP ratio to regulate cell polarity during migration
Source: Nat Commun. 2023 Dec 4;14:8003. doi: 10.1038/s41467-023-43645-w (PMC10695954; doi:10.1038/s41467-023-43645-w)
Supplement: Supplementary file 3 — Description of Additional Supplementary Files [file 41467_2023_43645_MOESM3_ESM.pdf]

Description of Additional Supplementary Files

File name: Supplementary Movie 1

Description: Ciliary cGMP buffering favours a cell polarity reversal phenotype in vitro. MGE-derived cortical interneuron co-electroporated with the cytoplasmic GFP construct and the mRFP-tagged 5HT6-GSponGee scavenger. A mRFP-positive PC is dynamically extended and retracted by the migrating interneuron as it migrates, as highlighted by the white arrowhead. Remarkably, the migrating cell undergoes a polarity reversal that is reflected by a reversal in the direction of migration. Time interval between frames, 5 minutes (3 frames per second).

File name: Supplementary Movie 2

Description: Ciliary cAMP buffering favours a cell polarity maintenance phenotype in vitro. MGE-derived cortical interneuron co-electroporated with the cytoplasmic GFP construct and the mRFP-tagged 5HT6-cAMP Sponge scavenger. A mRFP-positive PC is dynamically extended and retracted by the migrating interneuron as it migrates, as highlighted by the white arrowhead. The migrating cell maintains its polarity (successive translocation cycles occur within the same leading process), resulting in a highly directional migration behaviour. Time interval between frames, 5 minutes (3 frames per second).
